# The Advent of Generative Language Models in Medical Education

**DOI:** 10.2196/48163

**Published:** 2023-06-06

**Authors:** Mert Karabacak, Burak Berksu Ozkara, Konstantinos Margetis, Max Wintermark, Sotirios Bisdas

**Affiliations:** 1 Department of Neurosurgery Mount Sinai Health System New York, NY United States; 2 Department of Neuroradiology MD Anderson Cancer Center Houston, TX United States; 3 Department of Neuroradiology The National Hospital for Neurology and Neurosurgery University College London NHS Foundation Trust London United Kingdom

**Keywords:** generative language model, artificial intelligence, medical education, ChatGPT, academic integrity, AI-driven feedback, stimulation, evaluation, technology, learning environment, medical student

## Abstract

Artificial intelligence (AI) and generative language models (GLMs) present significant opportunities for enhancing medical education, including the provision of realistic simulations, digital patients, personalized feedback, evaluation methods, and the elimination of language barriers. These advanced technologies can facilitate immersive learning environments and enhance medical students' educational outcomes. However, ensuring content quality, addressing biases, and managing ethical and legal concerns present obstacles. To mitigate these challenges, it is necessary to evaluate the accuracy and relevance of AI-generated content, address potential biases, and develop guidelines and policies governing the use of AI-generated content in medical education. Collaboration among educators, researchers, and practitioners is essential for developing best practices, guidelines, and transparent AI models that encourage the ethical and responsible use of GLMs and AI in medical education. By sharing information about the data used for training, obstacles encountered, and evaluation methods, developers can increase their credibility and trustworthiness within the medical community. In order to realize the full potential of AI and GLMs in medical education while mitigating potential risks and obstacles, ongoing research and interdisciplinary collaboration are necessary. By collaborating, medical professionals can ensure that these technologies are effectively and responsibly integrated, contributing to enhanced learning experiences and patient care.

## Introduction

The rapid development of generative language models (GLMs) and artificial intelligence (AI) has ignited both excitement and concern in many fields, including medical education [1]. Sophisticated models such as OpenAI's ChatGPT [2] and Google's BARD [3] present opportunities to transform medical education with enhanced efficiency, interactivity, and realism. However, these new technologies also bring significant challenges and uncertainties.

The integration of these AI tools into medical education necessitates careful consideration and a nuanced understanding of potential implications. On the one hand, these models offer unparalleled capabilities, such as generating human-like text, simulating complex patient scenarios, and providing personalized learning experiences, thus fostering a more immersive and contextually relevant learning environment; on the other hand, potential issues of accuracy, reliability, misuse of AI-generated content, and academic integrity concerns are valid and demand careful deliberation. Additionally, the risk of bias, privacy issues, and potential dehumanization in the learning process call for caution. Another important aspect to consider is the “digital divide.” Unequal distribution of AI technology and resources could exacerbate existing disparities within the education system, particularly in low-resource settings and among disadvantaged student populations.

This viewpoint aims to explore these dimensions, discussing the benefits, challenges, ethical considerations, and academic integrity issues associated with incorporating AI into medical education. The objective is not to advocate for or against the use of AI in medical education but rather to provide an analysis that assists educators, practitioners, and policy makers in making informed decisions.

## Potential Benefits

GLMs hold immense potential in augmenting medical education through the generation of novel content, development of simulations, and creation of digital patients [4]. Compared to traditional computer-based simulations, these AI-enabled tools present a more dynamic and realistic learning experience. They offer more sophisticated scenarios for medical students to practice, thereby facilitating clinical decision-making and patient care [5]. By leveraging the advanced natural language understanding and generating capabilities of GLMs, platforms such as PerSim leverage them to provide students with contextually relevant patient scenarios that are more dynamic and adaptable than previous computer-based models [6]. The advantage of GLMs over these older models lies in their ability to generate unique and personalized responses, creating a more engaging and realistic interaction for the student. These enhanced capabilities permit the creation of immersive simulations and digital patients, which provide a more effective and individualized educational experience. These AI tools can provide real-time, individualized feedback based on a learner's performance and unique learning requirements during simulation exercises. This feedback can help students identify areas for improvement and refine their abilities. Furthermore, GLMs can generate customized simulation scenarios and case studies for each learner, allowing them to practice specific skills repeatedly in a controlled environment, thus fostering skill acquisition and refinement. In addition to benefiting students, these AI tools can also assist educators by providing resources and recommendations for simulation implementation. While human actors posing as simulated patients can offer a high degree of realism, AI-driven simulations provide a scalable, cost-effective alternative that can be customized to each student's learning needs. This innovative approach, thus, represents a significant advancement over traditional computer-based medical simulations.

AI-driven feedback and evaluation can help identify areas of weakness and improve overall performance [7]. The use of generative AI in formative and summative assessments in medical education can contribute to more personalized, efficient, and targeted evaluation methods. The creation of personalized quizzes for students is an illustration of the use of generative AI in medical education evaluations. By analyzing each student's strengths and weaknesses, generative AI can generate unique formative and summative assessments for each student. This could include a combination of questions focusing on areas in which the student needs improvement and topics in which the student excels, providing a more balanced and targeted evaluation of their medical knowledge. Furthermore, by analyzing student performance and providing real-time feedback, these AI-driven tools can help educators develop customized learning plans that address individual needs and improve overall outcomes.

As concrete examples of how AI and GLMs can impact medical education, one can consider the following scenarios. A medical educator can use a GLM to create a wide array of simulated patient scenarios. These scenarios can be highly realistic and varied, enabling students to gain exposure to a broad range of medical conditions and patient interactions. For instance, a medical student could interact with a simulated patient with a rare disease, ask questions, and receive responses that mimic real patient responses. This can allow the student to practice clinical reasoning skills in a safe and controlled environment. Likewise, medical researchers can use GLMs to scan and analyze vast amounts of medical literature quickly, identifying relevant studies and summarizing their findings. This can significantly reduce the time spent on literature reviews, allowing researchers to focus more on their primary research work.

AI-based educational resources not only cater to the needs of medical students but also aid in disseminating health-related information to the general public [8]. AI-based educational resources can provide patients with individualized health information, fostering health literacy and equipping people to make wise decisions regarding their health. Moreover, GLMs’ enhanced comprehension of complex medical terminology and context might enable AI-powered health companions such as Ada Health to provide more precise diagnostic suggestions and individualized health advice to both clinicians and patients [9]. 

The nuanced capabilities of these models to generate text at varying degrees of complexity could enhance the communication of health information. By adjusting the language and terminology used based on the intended audience, AI tools can make health information more accessible and understandable to a diverse range of individuals, from laypeople to medical professionals. This targeted communication approach can promote health literacy and empower individuals to make more informed decisions regarding their health.

One significant potential benefit of AI and GLMs in medical education that merits discussion is their potential to enhance machine translation, thereby fostering global collaboration and knowledge exchange. While machine translation is not a novel concept, the advent of AI and GLMs have significantly enhanced its accuracy and sophistication, making it a relevant point of discussion in the context of medical education. For instance, eBay's Machine Translation demonstrated a 7% increase in translation accuracy over its previous service [10], showcasing the potential of AI in overcoming language barriers. The implications of such advancements extend to medical education, where improved translation accuracy can foster global collaboration and knowledge exchange. AI-powered language models can translate medical lectures, webinars, and research articles in real time, making critical information accessible to individuals from diverse linguistic backgrounds. This can create a more inclusive learning environment and ensure that advancements in medical knowledge and patient care are globally accessible. Therefore, while machine translation itself is not new, the application of advanced GLMs promises a significant improvement over earlier models, and this potential benefit should not be overlooked.

## Challenges and Ethical Considerations

As GPT-4 continues to make waves in various industries, it is crucial to acknowledge the potential risks that come with AI integration. OpenAI’s Chief Executive Officer Sam Altman has highlighted the threat of widespread disinformation and cyberattacks as prominent concerns [11]. When it comes to integrating generative AI into medical education, these risks take on an even greater significance. Given the high stakes in health care and the potential for harm, the medical education field must be especially vigilant and proactive in managing these potential problems. The quality of the AI-generated content, for instance, is paramount. It requires meticulous assessment to ensure its accuracy and relevance. Measures such as proper prompting and iterative feedback loops can aid in enhancing the quality and reliability of AI-generated content in medical education [12,13].

Due to their training data, AI systems have been shown to exhibit discriminatory behavior and reinforce existing stereotypes. Incorporating GLMs into medical education necessitates exercising caution and addressing potential biases. Several past incidents—such as Microsoft's Tay chatbot tweeting racist and sexist content, and racial biases in facial recognition technology—demonstrate the need for vigilance [14,15]. By learning from these examples and avoiding potential pitfalls, we can develop more ethical and objective AI systems for medical education. To ensure the development of fair and responsible educational resources that promote accurate knowledge and uphold the integrity of the medical profession, it is essential to address inherent biases and ethical concerns. Recently, researchers have developed a logic-trained language model that significantly reduces harmful stereotypes by predicting relationships between sentences using context and semantic meaning [16]. This model outperforms large-scale models on logic-language comprehension tasks, demonstrating the potential for using logical learning to reduce bias and stereotypes in GLMs.

Finally, the incorporation of generative AI in medical education raises ethical and legal concerns, highlighting the need for AI ethics training for students to ensure the responsible and conscientious application of these advanced technologies [17]. Issues related to data privacy, transparency, and intellectual property must be addressed to ensure that these tools are used responsibly [18]. Furthermore, the potential manipulation of AI-generated content to produce misleading medical information or endorse unproven treatments could adversely impact not only medical students' education but also patients' understanding of their conditions. While AI can create highly realistic patient scenarios that can enhance medical education, it is crucial to note that these same tools can be misused or misrepresented. For example, an AI-generated scenario may be subtly altered to present incorrect or controversial medical advice or to favor a particular medical product or treatment. These altered scenarios, while appearing as realistic as accurate ones, could lead to confusion or misinterpretation of essential medical concepts, hence undermining the educational value and potentially harming patient care.

The unauthorized distribution of AI-generated content raises significant legal and ethical issues. This concern can be 2-fold. On the one hand, it pertains to the risks of sharing inappropriate content with AI models, such as uploading copyrighted material without obtaining the necessary permissions or exposing confidential patient information for training AI models—this is particularly problematic as these actions violate privacy laws and copyright regulations; on the other hand, it also concerns the potential for AI-generated content to inadvertently repeat copyrighted or confidential data that were used during its training phase. If an AI model were to generate and distribute content that mirrors confidential information or copyrighted material it was trained on, without proper acknowledgement or respect for privacy, it could have serious legal and ethical implications. Both these scenarios underscore the need for robust oversight, stringent data governance protocols, and clear usage policies when incorporating AI into medical education. The development of comprehensive guidelines and policies to govern the use of AI-generated content in medical education is crucial to ensure that its application in the learning process is both responsible and beneficial, preserving the integrity of medical education and the welfare of patients.

**Figure 1:**
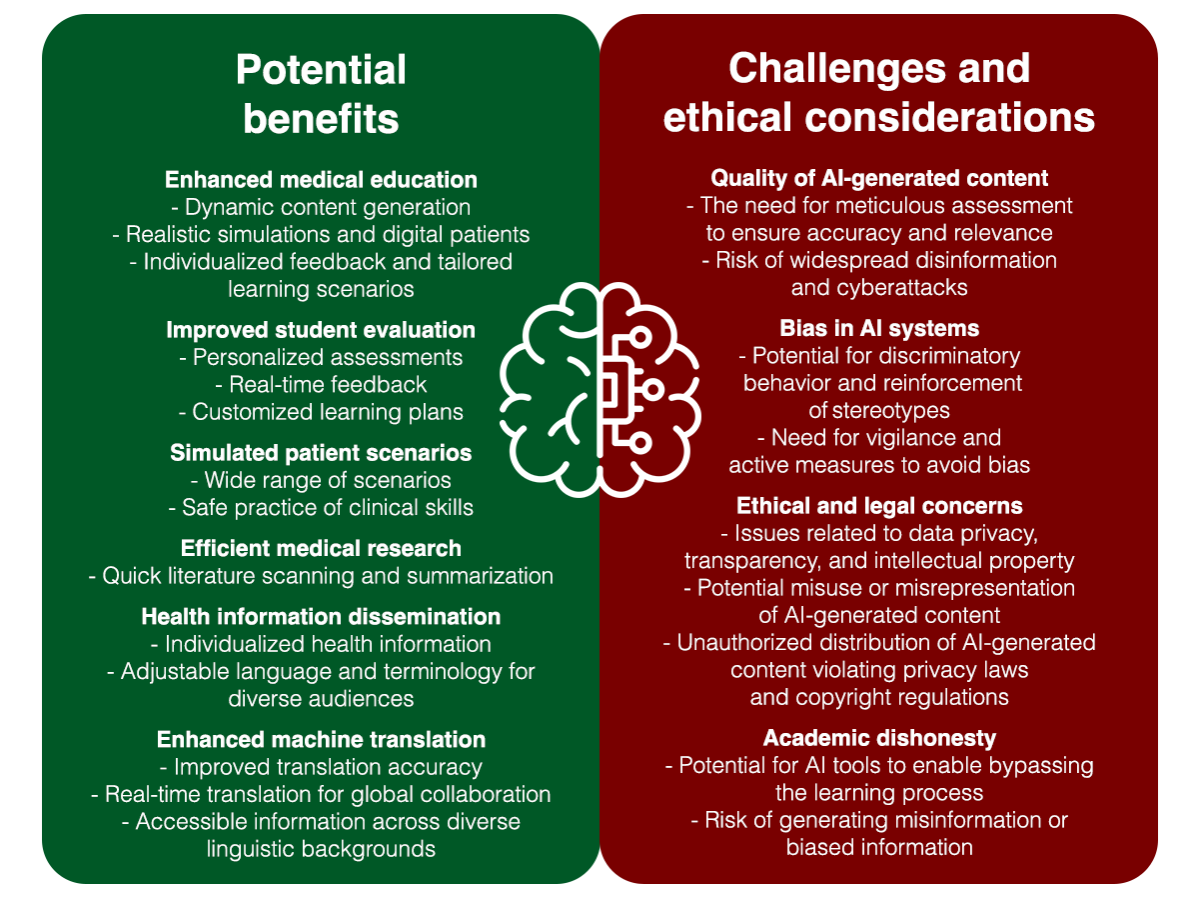
Potential benefits, challenges, and ethical considerations regarding the use of generative AI in medicine. AI: artificial intelligence.

Addressing the potential for AI-generated content to contribute to academic dishonesty is a critical issue [19,20]. The availability of GLMs could enable students to produce essays or assignment responses, bypassing the learning process and devaluing their educational experience. Further, AI-generated content can potentially produce misinformation or biased information, undermining trust in educational materials and leading to possible misinterpretation of essential medical concepts. To mitigate these concerns, academic institutions need to establish explicit guidelines concerning the use of AI-generated content in medical education. First, transparency is paramount. Students should be required to disclose their use of AI-generated content in their academic work. Equally, educators should also disclose their use of AI tools when developing educational materials, fostering a culture of transparency and setting an example for students. Second, the implementation of AI content detectors or AI classifiers is recommended, with the understanding that these tools are used not to detect plagiarism but to identify AI-generated content. However, the authors urge caution, as these detection tools are not always accurate or reliable, and the risk of unjust accusations of academic dishonesty is substantial. Therefore, these tools would need to undergo rigorous validation and regular updates to ensure their accuracy and fairness in determining the use of AI-generated content in student submissions. Third, while the potential of AI tools in education is highlighted in this paper, it is not meant to promote an unrestricted adoption of such technologies. Rather, the integration of AI into medical education should be carefully considered, and the use of AI-generated content should be limited to specific educational contexts, such as brainstorming or generating ideas for further research and discussion. Lastly, a shift toward diverse assessment methods is recommended. This could include presentations, practical assessments, and in-person written examinations, reducing the reliance on traditional essays that can be more easily generated by AI. By establishing, validating, and enforcing these guidelines, medical schools can promote ethical and responsible use of AI-generated content in their educational programs. Figure 1 summarizes the potential benefits, challenges, and ethical considerations regarding the use of generative AI in medical education.

## Future Directions and Perspectives

The future trajectory of medical education will be significantly influenced by the integration of GLMs and AI as these technologies continue to evolve [21]. The development of best practices, ethical principles, and regulations that support the responsible and effective use of AI in medical education hinges on the collective efforts of educators, researchers, and practitioners [22]. The creation of novel generative AI models specifically suited to medical education represents a promising area for future research. These models can produce accurate and pertinent content if they are trained on curated, high-quality data sets. In addition, effective interdisciplinary cooperation between computer scientists and medical professionals is necessary to develop AI-driven tools that cater to the particular requirements of medical education [23].

A critical consideration in this context is the accessibility of these data sets. Existing AI models are often trained on readily available data, which may not encompass specialized information necessary for advanced educational pursuits or rare diseases. Much of this vital information could be behind paywalls, posing a significant barrier to the development of competent AI models in these areas. Hence, future endeavors need to address the challenge of sourcing diverse and high-quality data sets for model training, ensuring that AI competency extends to niche and specialized areas of medical education.

The BLOOM project, a large language model created by over 1000 volunteer researchers, exemplifies the importance of transparency by sharing details about the data it was trained on, the challenges faced during development, and the methods used to evaluate its performance, while in contrast, the lack of transparency surrounding OpenAI's GPT-4 raises concerns as the company has not revealed any technical details about its development, data, computing power, or training techniques [24,25]. Transparency is also essential in medical education when developing and incorporating AI models. By openly sharing information about the data used for training, the challenges encountered, and the evaluation methods, developers can build trust and credibility within the medical community. This transparency allows medical professionals and educators to better understand the AI models' strengths and limitations, allowing them to make informed decisions about integrating these tools into their curriculum and practice.

The digital divide represents another crucial aspect to address when incorporating AI-driven resources into education [26,27]. As medical education gradually transitions from traditional printed materials toward digital AI-generated resources, it is of paramount importance to ensure equitable access to these resources. This involves considering disparities in access to technology and internet connectivity, particularly in low-resource settings such as rural or remote areas, institutions in transitional countries, or among students facing socioeconomic challenges.

Future research should prioritize the investigation of long-term effects of integrating generative AI into medical education. Understanding the impact of AI-driven tools on student learning, clinical judgment, and patient care outcomes is crucial for discerning potential advantages and drawbacks. Additionally, the creation of instructional materials and tutorials to aid educators in incorporating GLMs and AI into medical education could be invaluable. By sharing best practices and insights gleaned from early adopters, we can ensure that these technologies are used effectively, responsibly, and equitably.

## Conclusions

Incorporating GLMs and AI into medical education presents both opportunities and difficulties. GLMs can generate accurate, individualized content for students, allowing for more efficient learning experiences. To ensure the responsible application of these advanced technologies, it is necessary to address potential biases and ethical concerns. Educators, researchers, and practitioners must collaborate to create guidelines, policies, and best practices that promote the ethical and effective integration of GLMs and AI in medical education. In addition, for the medical community to develop trust and credibility, the development and implementation of AI-powered tools must be transparent. As the fields of AI and GLMs continue to develop, ongoing research and interdisciplinary collaboration will be essential to realizing their full potential in medical education while mitigating potential risks and obstacles.

## Abbreviations

AI: artificial intelligence
GLM: generative language model
